# High frequency electrical stimulation reduces α-synuclein levels and α-synuclein-mediated autophagy dysfunction

**DOI:** 10.1038/s41598-024-64131-3

**Published:** 2024-07-12

**Authors:** Jimmy George, Kashfia Shafiq, Minesh Kapadia, Lorraine V. Kalia, Suneil K. Kalia

**Affiliations:** 1 Toronto Western Hospital, Krembil Research Institute, University Health Network, 60 Leonard Avenue, Toronto, ON M5T 0S8 Canada; 2 Division of Neurology, Department of Medicine, Toronto Western Hospital, University Health Network, University of Toronto, Toronto, ON Canada; 3 Tanz Centre for Research in Neurodegenerative Diseases, University of Toronto, Toronto, ON Canada; 4 Division of Neurosurgery, Department of Surgery, Toronto Western Hospital, University Health Network, University of Toronto, Toronto, ON Canada; 5 KITE, University Health Network, Toronto, ON Canada; 6 CRANIA, Toronto, ON Canada

**Keywords:** Alpha-synuclein, ATPases, Autophagy, Deep brain stimulation, High frequency stimulation, Parkinson’s disease, Cell biology, Molecular biology, Neuroscience

## Abstract

Accumulation of α-synuclein (α-Syn) has been implicated in proteasome and autophagy dysfunction in Parkinson’s disease (PD). High frequency electrical stimulation (HFS) mimicking clinical parameters used for deep brain stimulation (DBS) in vitro or DBS in vivo in preclinical models of PD have been found to reduce levels of α-Syn and, in certain cases, provide possible neuroprotection. However, the mechanisms by which this reduction in α-Syn improves cellular dysfunction associated with α-Syn accumulation remains elusive. Using HFS parameters that recapitulate DBS in vitro, we found that HFS led to a reduction of mutant α-Syn and thereby limited proteasome and autophagy impairments due to α-Syn. Additionally, we observed that HFS modulates via the ATP6V0C subunit of V-ATPase and mitigates α-Syn mediated autophagic dysfunction. This study highlights a role for autophagy in reduction of α-Syn due to HFS which may prove to be a viable approach to decrease pathological protein accumulation in neurodegeneration.

## Introduction

Parkinson’s disease (PD) is a progressive neurodegenerative movement disorder characterized by the loss of dopaminergic neurons in the substantia nigra pars compacta (SNpc). A hallmark of PD pathology is the accumulation of misfolded forms of α-synuclein (α-Syn), including oligomers, smaller fibrillar species, and larger aggregates known as Lewy pathology^1,2^. Missense mutations in the *SNCA* gene that encodes α-Syn (e.g., A53T^3^, A30P^4^) manifest as autosomal dominant forms of familial PD. The mechanisms underlying α-Syn toxicity remain incompletely understood, but decreased degradation of α-Syn protein via protein clearance pathways, such as the ubiquitin proteosome system (UPS) and autophagy lysosome pathway (ALP)^5–9^, have been implicated as major contributors to its pathogenesis. Simultaneously, increased accumulation of α-Syn may deregulate the UPS^10–12^ or ALP^13,14^, further inhibiting its own degradation as well as clearance of its substrates and other proteins, yielding a vicious cycle^15^. Moreover, accumulated α-Syn has been shown to influence perturbations in several cellular organelles, including mitochondria, through events such as increased fragmentation and mitophagy^16–18^. These pathological mechanisms together cause accelerated neuronal death of dopaminergic neurons. Thus, α-Syn is considered a therapeutic target in PD with several strategies, such as enhancing its clearance, being actively pursued to modify disease progression.

Deep brain stimulation (DBS), which involves the neurosurgical implantation of electrodes to deliver high-frequency electrical stimulation (HFS) to deep nuclei in the basal ganglia or thalamus, provides relief from motor symptoms of PD with significant improvement in quality of life^19–21^. However, whether DBS has neuroprotective properties that delay progression of PD is still unknown^22^. Postmortem analysis of brain tissue from PD patients who received long‐term DBS targeting the subthalamic nucleus (STN-DBS) revealed no rescue of SNpc dopaminergic neurons or reduction in *α*‐Syn levels^23^. In contrast, STN-DBS in people with early-stage PD has been shown to slow the progression of tremor and reduces motor complications^24,25^. Preclinical studies using DBS in animal models have highlighted a possibility of its disease-modifying capability. STN-DBS mitigated the loss of SNpc dopaminergic neurons following the administration of the neurotoxins 6‐hydroxydopamine (6‐OHDA) in rats^26,27^ or 1‐methyl‐4‐phenyl‐1, 2, 3, 6‐tetrahydropyridine (MPTP) in non-human primates^28,29^. In an adeno-associated virus (AAV) model of PD, STN-DBS in rats overexpressing mutant A53T α-Syn reduced the loss of dopaminergic neurons in the SNpc and improved motor impairment^30^. Conversely STN‐DBS in a rat model overexpressing human wild-type (WT) α‐Syn failed to protect SNpc neuronal loss or alter motor phenotype ^31^. Our group recently demonstrated that DBS targeting the SNpc led to a reduction in α-Syn oligomers and total α-Syn levels in an AAV α-Syn model. Further, we found that HFS, mimicking DBS parameters, reduced WT α-Syn oligomers and mutant α-Syn levels in primary cortical neurons^32^, suggesting that HFS can reduce pathological forms of α-Syn, although via unknown mechanisms. Recent studies simulating transcranial direct current stimulation (tDCS) in neurotoxin-based cell culture^33^ and animal^34^ models implicate the macroautophagy pathway in clearance of α-Syn in response to neuromodulation. With the ability of stimulation to target autophagy, it is conceivable that the α-Syn reduction we observed with HFS could be mediated by a degradation pathway. Thus, we set out to uncover the effects of HFS on the major cellular degradation pathways associated with α-Syn accumulation.

## Material and methods

### SHSY5Y cell culture and transfection

SH-SY5Y cells (ATCC, CRL-2266) were cultured in Dulbecco’s Modified Eagle Medium (DMEM)/F12 medium (Sigma) supplemented with 10% (w/v) fetal bovine serum (Life Technologies) and 1% antibiotic–antimycotic (Life Technologies). The cells were maintained at 37 °C in a humidified atmosphere of 5% (v/v) CO_2_. Cells were grown to 50% confluence in 24-well culture dishes and then transfected with pcDNA, A53T α-synuclein, Ub^G76V^-GFP, and mito-QC plasmids using Lipofectamine 2000 (Thermo Fisher Scientific) according to manufacturer’s protocol.  To probe for ALP involvement, cells were treated with Bafilomycin A1 (40 nM) or DMSO (vehicle) for 6 h. For investigate UPS contribution, cells were treated with MG132 (10 µM) for 6 h, followed by stimulation. The cells were then fixed using 4% paraformaldehyde (PFA) for immunofluorescence staining or lysed for immunoblotting using RIPA.

### Adeno-associated viruses

Adeno-associated virus (AAV) of a 1/2 serotype, under the CAG promotor, a hybrid of chicken beta actin (CBA) promotor fused with the cytomegalovirus (CMV) immediate early enhancer sequence, was used to express A53T α-synuclein (AAV-A53T) (2.55 × 10^12^ genomic particles (gp) per ml; Genedetect Ltd). For an empty vector control (AAV-EV), an AAV 1/2 vector lacking the A53T α-synuclein open reading frame was used. AAV1/2 vector expressing Venus YFP alone (AAV-YFP) was used as an internal control for co-transduction with AAV-A53T or AAV-EV. To probe mitophagy dysfunction, mCherry-EGFP-FIS1 (mito-QC) open reading frame was expressed using an AAV serotype 8 under control of the CAG promotor (2.18 × 10^12^ gp; Vector Builder Inc.).

### Primary neuron culture and AAV transduction

Pregnant Sprague–Dawley rats (E17) were purchased from Envigo. Embryos were surgically removed, and cortices were dissected in Hanks Balanced salt solution (Gibco). The meninges were removed, and cells were dissociated using a papain dissociation system (Worthington) before being resuspended in Neurobasal medium A supplemented with antibiotic–antimycotic solution (Gibco), L-glutamine substitute (GlutaMAX™; Gibco), and factor B27 (Gibco).

Neurons were plated on poly-D-lysine coated glass coverslips at a density of 5 × 10^5^ cells/well or on poly-D-lysine coated 6-well cell culture plates at a density of 2 × 10^6^ cells/well and incubated at 37 °C in 5% CO_2_ with half media changes every 3 days. Neurons were transduced with the experimental AAVs (AAV-A53T, AAV-EV, AAV-YFP, AAV-mito-QC) 2 days post-isolation at a multiplicity of infection of 3000. Media containing AAV vectors were removed after 72 h and replaced with fresh media 5 days post-isolation and stimulated. Following stimulation protocol (see below), neurons were immediately fixed with 4% PFA for immunofluorescence staining or lysed for immunoblotting.

### High frequency electrical stimulation

Transfected SH-SY5Y cells and transduced primary cortical neurons were stimulated using an IonOptix C-Pace EM multichannel stimulator configured with a 6-well C-Dish fitted with carbon electrodes. An external signal was generated using a waveform generator (Agilent 33220A, Keysight Technologies) with the following parameters: 120 Hz, 5 V, 0.4 ms pulse width, for a duration of 2 h. HFS of neurons was initiated in fresh media immediately following 72 h of AAV treatment on DIV5 (days in vitro) and fixed in 4% PFA at the end of the 2-h stimulation session. All immunofluorescence outcomes secondary to HFS were generated from 3 independent cell dissociations (n = 3) with 10 neurons or cells analysed per experiment. SH-SY5Y cells and cortical neurons were placed in the cell culture incubator during stimulation, with the IonOptix system placed outside the incubator.

### Real-time quantitative RT-PCR

Total RNA was isolated from SH-SY5Y cells using RNeasy Mini Kit (50) (Qiagen, Cat#74,104) according to the manufacturer’s instructions. RNA quantity and purity were determined by spectrophotometric analysis (NanoDrop 2000c; ThermoFisher Scientific). One microgram of purified RNA was reversely transcribed to cDNA using random primer (Invitrogen) and Superscript™ Ш Reverse Transcriptase (Invitrogen Cat# 18,080,044). Quantitative real-time PCR was performed in the Quant Studio™ 5 Real-Time PCR System (ThermoFisher Scientific) using Power Track™ SYBR Green Master Mix (Invitrogen, Cat# A46109). The thermal cycling conditions were as follows: amplifications were performed starting at 95 °C for 2 min, followed by 40 cycles of 95 °C for 15 s and 60 °C for 1 min. Melting curve analysis began at 95 °C for 15 s, followed by at 60 °C for 1 min and at 95 °C for 15 s. Specificity of the product amplification was confirmed by melting curve analysis. Primers for the target human gene α-Syn were forward- 5’- CCAAAGAGCAAGTGACAAATGTTG -3’, reverse- 5’- CCTCCACTGTCTTCTGGGCTACT -3’. Primers for the house keeping human gene GAPDH were forward- 5’- GTCTTCACCACCATGGAGAA -3’, reverse- 5’- ATCCACAGTCTTCTGGGTGG -3’.

### Antibodies

The following primary antibodies were used: anti-α-Syn (Invitrogen; 211 32–8100), anti-GFP (Cell Signalling; 2956), anti-LAMP-1 (Cell Signaling; 9091S), anti-P62 (Cell Signalling; 5114S), anti-TOMM20 (Abcam; EPR15581-39), anti-LC3B (Cell Signalling; 3868S), anti-GAPDH (Cell Signalling; 2118S), anti-ATP6V0C (Invitrogen; PA5-23,972). Secondary antibodies used included: horseradish peroxidase (HRP) linked anti-rabbit IgG (Cell signalling; 7074S), HRP linked anti-mouse IgG (Cell Signalling; 7076P2), donkey anti-mouse 647 (Invitrogen; A31571), and goat anti-mouse 488 (Invitrogen; A11008).

### Immunofluorescence staining of cultured cells and neurons

Post fixation using 4% PFA, cells and neurons were permeabilized with 0.2% Triton X-100 for 15 min, washed three times with PBS and then incubated with blocking solution (1% BSA, 22.52 mg/mL glycine, 0.1% Tween-20 in PBS) for one hour, followed by overnight incubation with primary antibodies diluted in blocking solution at 4 °C. Primary antibodies were washed off with PBS, and cells were incubated in secondary antibodies diluted in blocking solution. Secondary antibodies were washed off with PBS. The coverslips were mounted on slides using ProLong TM Gold antifade Mountant with DAPI (ThermoFisher).

### Western blot analysis

Cell lysates were scraped and pelleted by centrifugation at 2350 × *g* for 5 min at 4 °C. The supernatant was discarded, and the pellet was lysed in RIPA buffer (50 mM Tris–HCl pH 7.4, 1% Nonidet P-40, 150 mM NaCl, 1 mM EDTA) supplemented with protease (cOmplete™ ULTRA Tablets, Mini, EASYpack Protease Inhibitor Cocktail, Roche) and phosphatase (PhosSTOP EASYpack, phosphatase inhibitor tablets, Roche) inhibitors and kept on ice for 30 min. Following incubation, samples were centrifuged at 17,100 × *g* for 20 min at 4 °C. The insoluble pellet was discarded, and the supernatant was collected for subsequent analysis. Protein concentration in RIPA-soluble samples was quantified using the DC protein assay (BioRad). 4X sodium dodecyl-sulfate polyacrylamide gel electrophoresis (SDS-PAGE) sample buffer was added to the whole cell lysate and boiled for 10 min at 95 °C. Samples were subsequently run on a 4–20% Mini-Protean TGX Precast gel (Bio-Rad). The gel was transferred onto polyvinylidene fluoride membrane (Bio-Rad) and blocked at room temperature in 5% w/v milk in 0.1% TBS-T for 1 h and incubated in primary antibodies overnight at 4 °C. The PVDF membrane was washed the next day with PBS-T and incubated in HRP-conjugated secondary antibodies and detected using enhanced chemiluminescence (Pierce).

### Imaging and quantification

Confocal images were captured with a Zeiss LSM880 confocal microscope. SH-SY5Y cells and cortical neurons were imaged at 63X magnification with additional 2X zoom at 405 nm, 488 nm, 555 nm, and 639 nm laser lines. Z-stacks were taken within linear range at a constant gain for each channel at a 920 × 920 pixel-ratio. The software was programmed to acquire an image every ~ 0.5 μm, capturing all the cells visible in the z-plane in each field of view using a 63X oil objective. Fluorescence intensity was calculated using the Surface module, while puncta size was calculated using the Spots module in IMARIS (Oxford instruments). For quantification of the number of mito-lysosomes per cell and neuron, contours were manually drawn around the cell body and processes of SH-SY5Y cells and cortical neurons to define the region of interest (ROI). Using the IMARIS spots module, puncta were selected in both the mCherry and EGFP channels at an approximate size of 0.4 μm diameter. The ‘co-localize spots’ function was used to measure the number of mCherry puncta that were not co-localized with EGFP puncta. For quantification of the percentage of LC3B co-localized with TOMM20, contours were manually drawn around cells to define the ROI. Using IMARIS, the spots module was used to identify LC3B, and the surfaces module was used to identify TOMM20. The ‘spots close to surface’ function was used to measure the number of LC3B puncta that were and were not co-localized with TOMM20. The percentage of LC3B co-localized with TOMM20 was calculated by dividing the number of co-localized LC3B spots by the total number of LC3B spots.

### Statistical analysis

Statistical analysis was performed using GraphPad Prism 8. The specific statistical tests performed are indicated. All data are represented as mean ± s.e.m. with 3 independent experiments. One-way ANOVA or Student t-test was performed as indicated.

### Ethics approval

The University Health Network Animal Care Committee approved all animal procedures in accordance with the guidelines and regulations set by the Canadian Council on Animal Care. Experiments were performed in compliance with the Animal Research: Reporting of In Vivo Experiments (ARRIVE) guidelines.

## Results

### High frequency electrical stimulation causes transient reduction in mutant α-Syn levels

**Figure 1:**
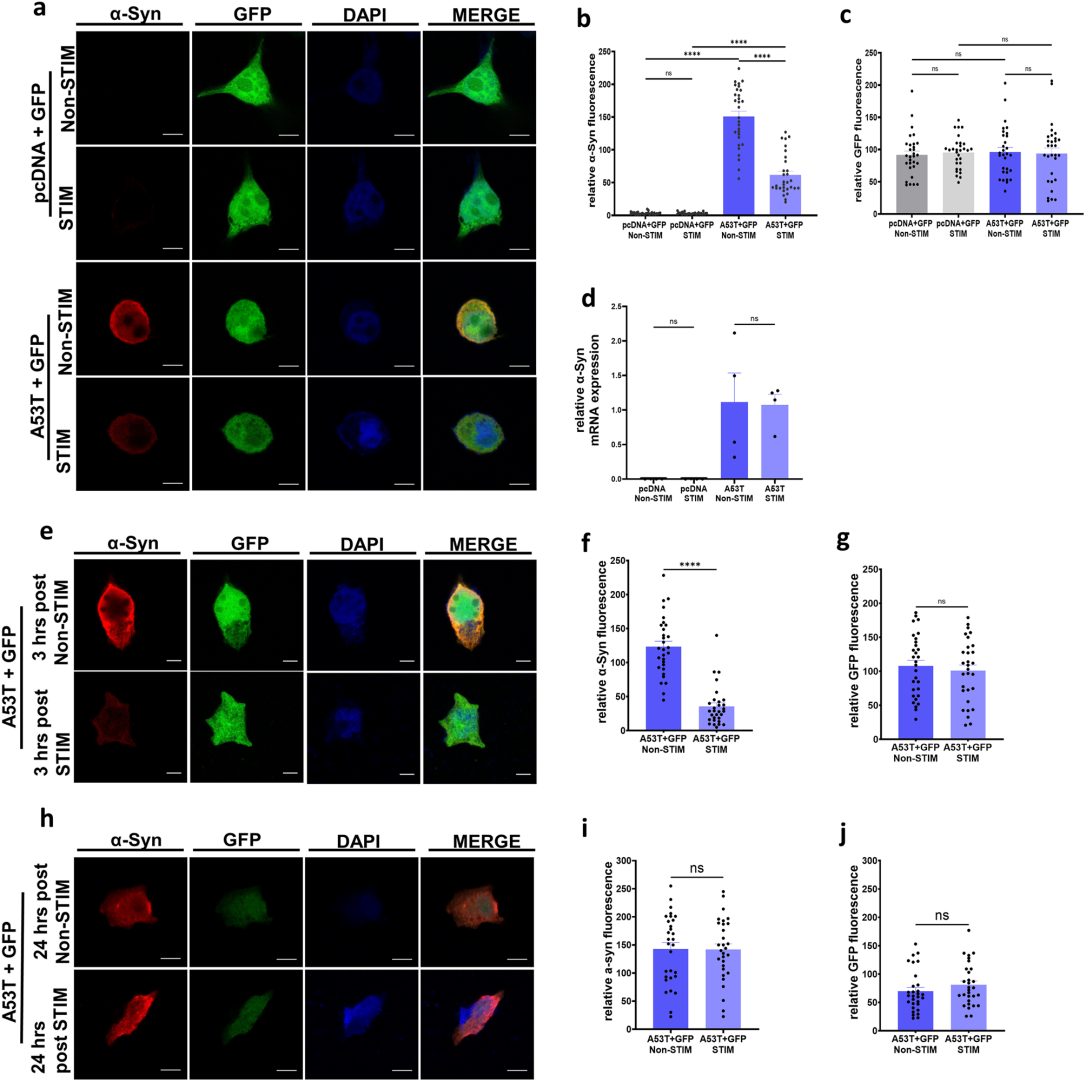
High frequency electrical stimulation causes transient reduction in mutant α-Syn levels (**a**) Representative images of SH-SY5Y cells co-transfected with GFP (green fluorescent protein) and A53T or pcDNA after stimulation for 2 h and non-stimulated controls, stained with DAPI (blue), α-Syn (red) and GFP (green) (Scale bars 5 µm) (**b**) Quantification of α-Syn fluorescence in SH-SY5Y cells after stimulation for 2 h, compared with non-stimulated controls. n = 3 independent experiments, analyzing 10 cells per condition. Bars represent mean ± s.e.m., ****p < 0.0001, ns non-significant, one-way ANOVA, Tukey’s m.c.t. (**c**) Quantification of GFP levels. n = 3 independent experiments, analyzing 10 cells per condition.  Bars represent mean ± s.e.m., ns non-significant, one-way ANOVA, Tukey’s m.c.t. (**d**) Quantification of α-Syn mRNA levels from A53T or pcDNA transfected SH-SY5Y cells after stimulation compared with non-stimulated controls. n = 4 independent experiments. Bars represent mean ± s.e.m., ns non-significant, one-way ANOVA, Tukey’s m.c.t. (**e**) Representative images of SH-SY5Y cells co-transfected with GFP and A53T or pcDNA after recovery of 3 h post stimulation compared with non-stimulated controls stained with DAPI (blue), α-Syn (red) and GFP (green) (Scale bars 5 µm) (**f**) Quantification of α-Syn fluorescence in SH-SY5Y cells after 3 h recovery post stimulation and non-stimulated controls. n = 3 independent experiments, analyzing 10 cells per condition. Bars represent mean ± s.e.m., ****p < 0.0001, two-tailed unpaired t-test (**g**) Quantification of GFP fluorescence levels in SH-SY5Y cells after 3 h recovery post stimulation and non-stimulated controls. n = 3 independent experiments, analyzing 10 cells per condition. Bars represent mean ± s.e.m., ns non-significant, two-tailed unpaired t-test. (**h**) Representative images of SH-SY5Y cells co-transfected with GFP and A53T after 24 h recovery post stimulation compared with non-stimulated controls stained with DAPI (blue), α-Syn (red) and GFP (green) (Scale bars 5 µm) (**i**) Quantification of α-Syn fluorescence in SH-SY5Y cells after 24 h recovery post stimulation and non-stimulated controls. n = 3 independent experiments, analyzing 10 cells per condition. Bars represent mean ± s.e.m., ns non-significant, two-tailed unpaired t-test. (**j**) Quantification of GFP fluorescence levels. n = 3 independent experiments, analyzing 10 cells per condition. Bars represent mean ± s.e.m., ns non-significant, two-tailed unpaired t-test.

We previously demonstrated that HFS reduced pathological α-Syn levels in cultured neurons^32^. Here we aimed to determine if similar modulation can be achieved in other cell types to facilitate dissection of the underlying mechanisms. To achieve this, we used human SH-SY5Y cells, an immortalized cell line routinely used in cell work for PD research^35,36^. SH-SY5Y cells were co-transfected with human A53T α-Syn or pcDNA with green fluorescent protein (GFP) as an internal control to assess for non-specific effects of HFS*.* We electrically stimulated the cells for 2 h using the IonOptix C-Pace EM Culture Pacing System with settings mimicking clinical DBS^32^ and fixed immediately for immunofluorescent analysis (Fig. 1a). We observed that HFS did not alter levels of endogenous α-Syn based on relative fluorescence intensity (RFU) assessment (3.1 ± 0.27 RFU stimulated *vs* 3.2 ± 0.3 RFU non-stimulated). In contrast, HFS led to a partial reduction of mutant α-Syn in SH-SY5Y cells transfected with A53T α-Syn compared to non-stimulated cells transfected with A53T α-Syn (61.4 ± 5.67 RFU stimulated *vs* 150.8 ± 8.2 RFU unstimulated, ****p < 0.0001, one-way ANOVA, Tukey’s m.c.t.) (Fig. 1b). This decrease was still significantly higher than stimulated pcDNA-transfected control cells (3.1 ± 0.27 RFU) (Fig. 1b). No differences in GFP fluorescence intensity were noted (109.1 ± 7.45 RFU stimulated *vs* 96.2 ± 7.12 RFU non-stimulated) (Fig. 1c), demonstrating the specificity of HFS on mutant α-Syn levels. We also found that stimulated and non-stimulated A53T α-Syn transfected cells exhibited similar α-Syn mRNA levels (1.07 ± 0.15 relative mRNA levels stimulated *vs* 1.115 ± 0.42 relative mRNA levels non-stimulated) (Fig. 1d), indicating that the decreased α-Syn levels were not caused by HFS modulation of α-Syn transcription but due to the alteration of protein levels. Taken together our results suggest that HFS does not inhibit the expression of α-Syn but promotes the degradation of accumulated protein.

To evaluate the duration of this HFS effect, transfected SH-SY5Y cells stimulated for 2 h were allowed to recover in the incubator post-stimulation for 3 h (Fig. 1e) and 24 h (Fig. 1h), before being fixed for immunofluorescence. The reduction in A53T α-Syn levels following HFS was preserved 3 h after the end of stimulation (35.45 ± 5.307 RFU stimulated *vs* 123.4 ± 7.98 RFU non-stimulated, ****p < 0.0001 unpaired t-test) (Fig. 1f), with no differences in GFP fluorescence intensity (93.78 ± 8.51 RFU stimulated *vs* 96.21 ± 7.12 RFU non-stimulated) (Fig. 1g). Interestingly, the HFS effect was no longer  observed in cells allowed to recover for 24 h post stimulation (141.7 ± 10.18 RFU stimulated *vs* 142.9 ± 11.27 RFU non-stimulated) (Fig. 1i), along with no difference in GFP levels (81 ± 6.80 RFU stimulated *vs* 69.6 ± 6.62 RFU non-stimulated) (Fig. 1j). Taken together, these findings suggest that short-term HFS, mimicking DBS parameters, yields a transient reduction in the protein levels of mutant α-Syn.

### α-Syn reduction due to high frequency electrical stimulation limits UPS dysfunction

The ubiquitin proteosome pathway mediates covalent tagging of multiple ubiquitin molecules to protein substrates, followed by degradation of the tagged proteins by the 26S proteasome complex, and then the release of free and reusable ubiquitin^37^.  In vitro studies suggest that overexpression of WT α-Syn^38^ or mutant α-Syn^39,40^ inhibits proteasome activity in cells, which could impair degradation of protein substrates such as α-Syn. Recently, our group demonstrated that A53T α-Syn overexpression in the rat SNpc leads to catalytic impairment of the 26S proteasome and UPS dysfunction that precedes the loss of dopaminergic neurons and associated motor deficits^12^. Thus, we set out to determine if HFS has any impact on α-Syn-mediated UPS dysfunction.

**Figure 2:**
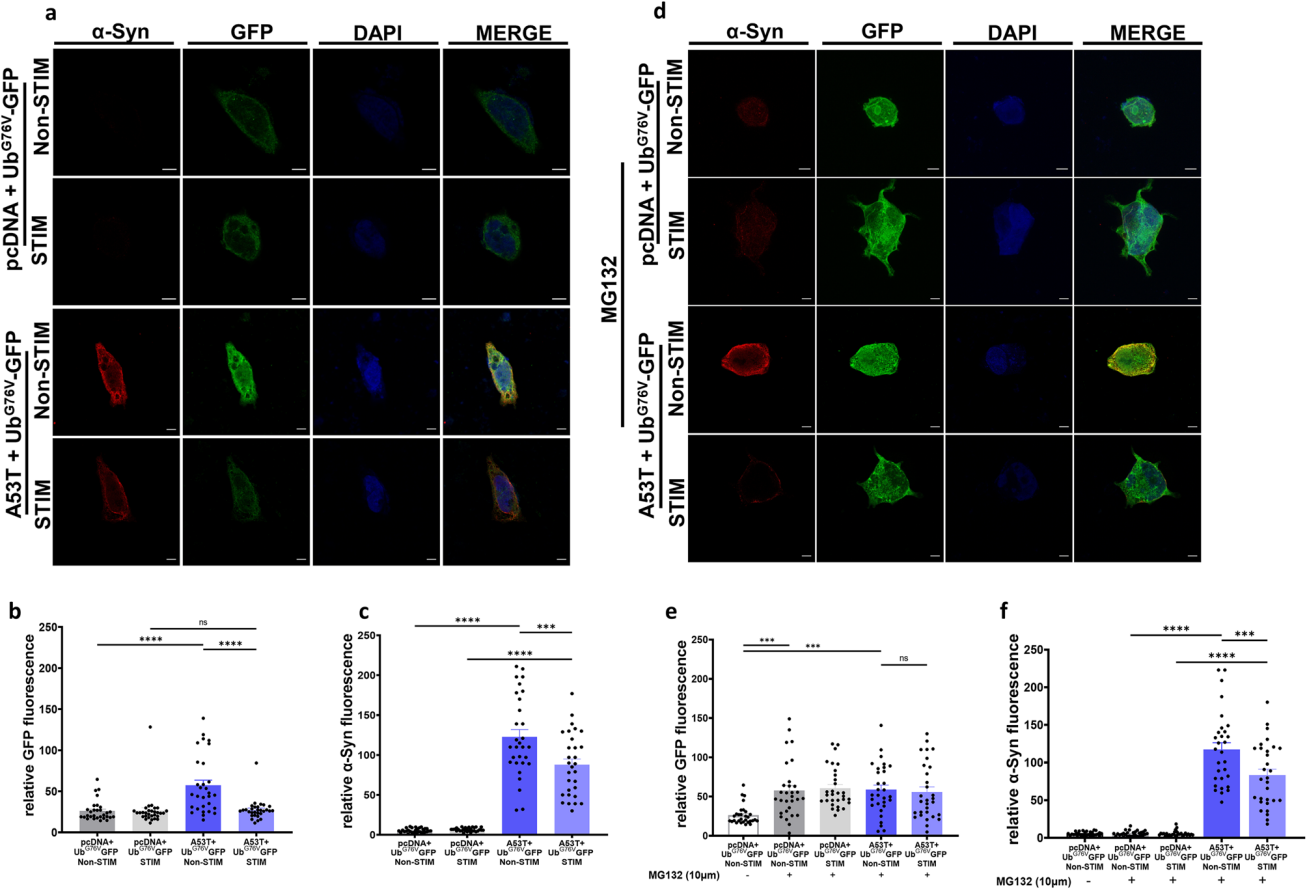
α-Syn reduction due to high frequency electrical stimulation limits UPS dysfunction. (**a**) Representative images of SH-SY5Y cells co-transfected with Ub^G76V^-GFP and A53T or pcDNA after stimulation for 2 h and non-stimulated controls, stained with DAPI (blue), α-Syn (red) and anti-GFP (green) (Scale bars 5 µm) (**b**) Quantification of GFP fluorescence levels depicting Ub^G76V^ levels in cells transfected with A53T and pcDNA controls in presence and absence of stimulation n = 3 independent experiments, analyzing 10 cells per condition. Bars represent mean ± s.e.m., ****p < 0.0001, ns non-significant. one-way ANOVA, Tukey’s m.c.t. (**c**) Quantification of α-Syn fluorescence in SH-SY5Y cells after stimulation for 2 h and non-stimulated controls. n = 3 independent experiments, analyzing 10 cells per condition. Bars represent mean ± s.e.m., ***p < 0.001, ****p < 0.0001 one-way ANOVA, Tukey’s m.c.t. (**d**) Representative images of SH-SY5Y cells co-transfected with Ub^G76V^-GFP and A53T or pcDNA, treated with MG132, after stimulation for 2 h and non-stimulated controls, stained with DAPI (blue), α-Syn (red) and anti-GFP (green) (Scale bars = 5 µm) (**e**) Quantification of GFP fluorescence levels. n = 3 independent experiments, analyzing 10 cells per condition. Bars represent mean ± s.e.m., ***p < 0.001, ns non-significant, Tukey’s m.c.t. (**f**) Quantification of α-syn fluorescence levels. n = 3 independent experiments, analyzing 10 cells per condition. Bars represent mean ± s.e.m., ***p < 0.001, ****p < 0.0001, one-way ANOVA, Tukey’s m.c.t.

To achieve this, we used the ubiquitin reporter protein Ub^G76V^-GFP to measure UPS function in the context of mutant A53T α-Syn overexpression in stimulated and non-stimulated cells (Fig. 2a)^12,41^. The Ub^G76V^-GFP  vector contains a ubiquitin fusion degradation signal consisting of an N-terminally linked ubiquitin moiety which accepts polyubiquitin chains linked through Lys48 and Lys29 linkages^12,42^. A  reduction in clearance of polyubiquitinated substrates by the 26S proteasome causes an accumulation of Ub^G76V^-GFP, which is detected by native fluorescence or immunofluorescent staining with anti-GFP antibodies^12,42^. SH-SY5Y cells co-transfected with A53T α-Syn and Ub^G76V^-GFP exhibited increased GFP fluorescence compared to cells co-transfected with pcDNA and Ub^G76V^-GFP indicative of UPS dysfunction secondary to α-Syn overexpression (57.1 ± 6.37 RFU A53T + Ub^G76V^-GFP non-stimulated *vs* 26.09 ± 2.23 RFU pcDNA + Ub^G76V^-GFP non-stimulated, ****p < 0.0001, one-way ANOVA, Tukey’s m.c.t.) (Fig. 2b). GFP levels were unaltered by HFS with no difference between stimulated pcDNA-Ub^G76V^-GFP cells and non-stimulated pcDNA-Ub^G76V^-GFP cells (26.47 ± 3.636 RFU stimulated *vs* 26.09 ± 2.23 RFU non-stimulated) (Fig. 2b). However, when cells co-transfected with A53T α-Syn and Ub^G76V^-GFP were stimulated, we noted a decrease in the levels of GFP fluorescence when compared to non-stimulated A53T-Ub^G76V^-GFP cells (27. 38 ± 2.24 RFU stimulated *vs* 57.1 ± 6.37 RFU non-stimulated, ****p < 0.0001, one-way ANOVA, Tukey’s m.c.t.) (Fig. 2b), in addition to reduced α-Syn levels (87.87 ± 7.14 RFU stimulated *vs* 122.7 ± 9.21 RFU non-stimulated) (Fig. 2c). These findings suggest an improvement of UPS function associated with the reduction of α-Syn due to HFS.

Although not yet completely elucidated, α-Syn has been shown to be degraded by the UPS which is impaired in PD^12,41,43^. Thus, it is possible that the reduction of α-Syn is due to HFS-mediated enhancement of UPS activity. To resolve this, we used the pharmacological proteasome inhibitor MG132 (Fig. 2d)^12,44–46^. Treating SH-SY5Y cells with MG132 yielded an increase in GFP fluorescence levels compared to untreated pcDNA controls (57.54 ± 6.5 RFU MG132 pcDNA *vs*. 26.09 ± 2.23 RFU non-treated pcDNA. ***p < 0.001, one-way ANOVA, Tukey’s m.c.t.) (Fig. 2e), indicating UPS dysfunction. However, we found no difference in GFP fluorescence levels between stimulated and non-stimulated MG132 treated pcDNA transfected cells (60.5 ± 4.7 RFU stimulated *vs* 57.5 ± 6.54 RFU non-stimulated) (Fig. 2e), indicating that HFS did not affect UPS dysfunction caused by MG132 proteosome inhibition. Moreover, stimulation of cells co-transfected with A53T α-Syn and Ub^G76V^-GFP and treated with MG132 still demonstrated a decrease in the levels of α-Syn compared to non-stimulated cells (83.4 ± 7.75 RFU stimulated *vs* 117.4 ± 8.93 RFU, ***p < 0.001, one-way ANOVA, Tukey’s m.c.t.) (Fig. 2f) despite no reduction in GFP fluorescence levels (55.78 ± 6.53 RFU stimulated *vs* 58.7 ± 5.88 RFU non-stimulated) (Fig. 2e). Taken together, we surmise that α-Syn reduction due to HFS facilitates recovery of UPS dysfunction.

### High frequency stimulation partially rescues α-Syn-mediated autophagy impairment

Macroautophagy is one of the major degradation pathways in cells and plays a pivotal role in maintaining effective turnover of proteins and damaged organelles. Accumulation of pathological proteins may tax the ALP causing its dysfunction. Mutant forms of α-Syn, such as A53T and A30P, bind to lysosomes but are poorly internalized and act as uptake blockers, preventing their own uptake and degradation by lysosomes. This contributes to a vicious cycle of further accumulation^47^ and increased autophagic impairment^48–51^.

**Figure 3:**
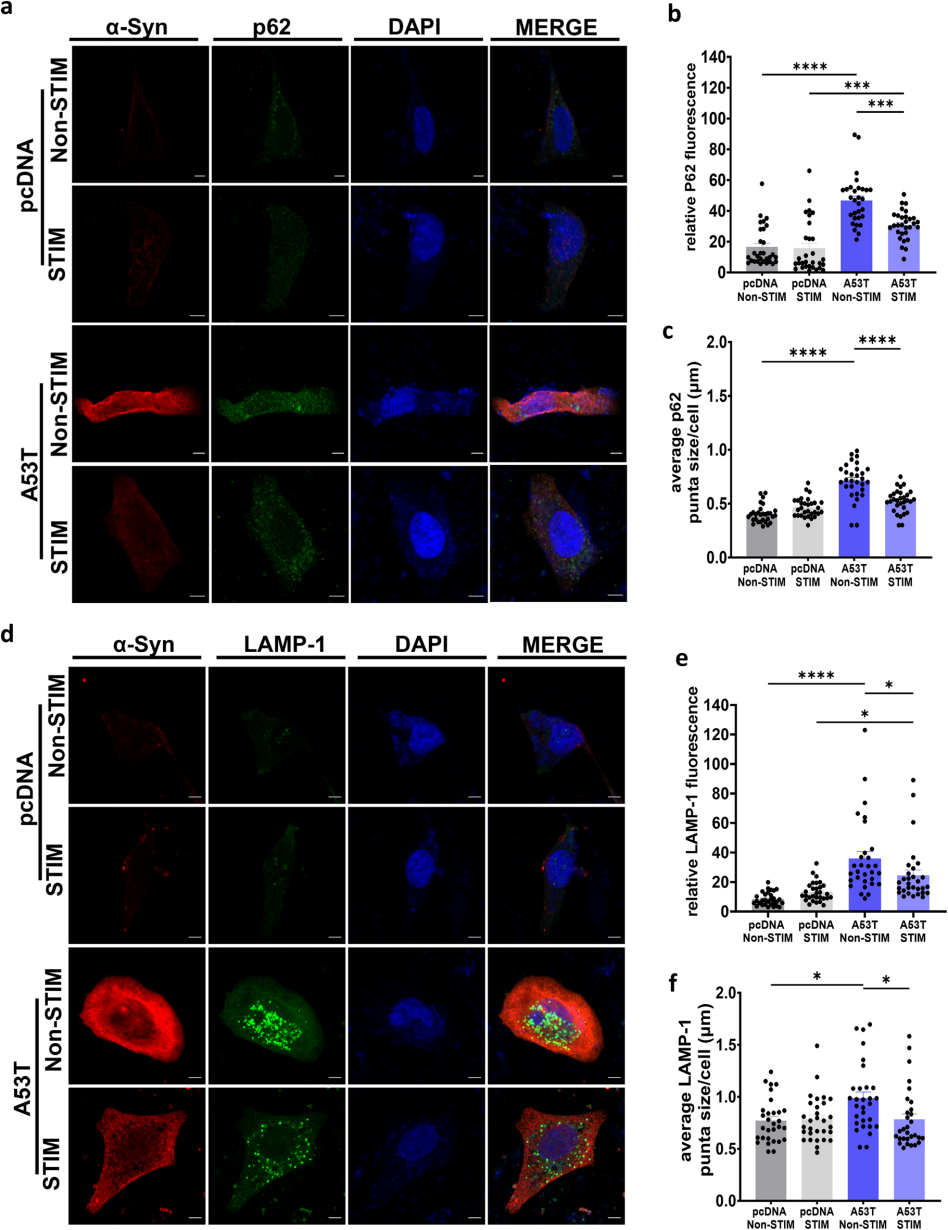
High frequency stimulation partially rescues α-Syn-mediated autophagy impairment. (**a**) Representative images of SH-SY5Y cells transfected with A53T or pcDNA after stimulation for 2 h and non-stimulated controls, stained with DAPI (blue), α-Syn (red) and P62 (green) (Scale bars = 10 µm) (**b**) Quantification of P62 fluorescence. n = 3 independent experiments, analyzing 10 cells per condition. Bars represent mean ± s.e.m., ***p < 0.001, ****p < 0.0001, one-way ANOVA, Tukey’s m.c.t. (**c**) Quantification of P62 average puncta size. n = 3 independent experiments, analyzing 10 cells per condition. Bars represent mean ± s.e.m., ****p < 0.0001, one-way ANOVA, Tukey’s m.c.t. (**d**) Representative images of SH-SY5Y cells transfected with A53T or pcDNA after stimulation for 2 h and non-stimulated controls, stained with DAPI (blue), α-Syn (red) and LAMP-1 (green (Scale bars = 10 µm) (**e**) Quantification of LAMP-1 fluorescence. n = 3 independent experiments, analyzing 10 cells per condition. Bars represent mean ± s.e.m., *p < 0.05, ****p < 0.0001, one-way ANOVA, Tukey’s m.c.t. (**f**) Quantification of LAMP-1 average puncta size. n = 3 independent experiments, analyzing 10 cells per condition. Bars represent mean ± s.e.m., *p < 0.05, one-way ANOVA, Tukey’s m.c.t., ns non-significant.

To investigate whether HFS-mediated α-Syn reduction not only limits UPS dysfunction but also affects autophagic impairment, we examined the levels of P62, a selective cargo receptor for autophagy and a widely used marker for autophagic flux and autophagosome formation^52^. P62 is a main receptor enabling crosstalk between the UPS and ALP with inhibition of UPS causing excess ubiquitinated products to be transported to the autophagosome via binding with P62 and others^52–54^. Along with P62 (Fig. 3a), we also measured the levels of Lysosomal Associated Membrane Protein 1 (LAMP1) (Fig. 3d), a marker of late endosomes and lysosomes. Alterations in P62 and LAMP-1 levels  are often associated with autophagy dysfunction^55–57^. We observed that HFS of A53T α-Syn cells demonstrated significantly reduced P62 fluorescence levels compared to non-stimulated controls (30.81 ± 1.69 RFU stimulated *vs* 46.6 ± 2.8 RFU non-stimulated, ***p < 0.001, one-way ANOVA, Tukey’s m.c.t.) (Fig. 3b). HFS also reduced the average size of P62 puncta compared to non-stimulated A53T α-Syn transfected cells (0.523 ± 0.019 µm stimulated *vs* 0.71 ± 0.03 µm non-stimulated, ****p < 0.0001, one-way ANOVA, Tukey’s m.c.t.) (Fig. 3c). Similar to P62 levels, LAMP-1 levels were significantly reduced by HFS (24.62 ± 3.5 RFU stimulated *vs* 35.98 ± 4.6 RFU non-stimulated, *p < 0.05, one-way ANOVA, Tukey’s m.c.t.) (Fig. 3e). Furthermore, LAMP-1 puncta size was reduced by HFS (0.78 ± 0.05 µm stimulated *vs* 0.98 ± 0.05 µm non-stimulated, *p < 0.05, one-way ANOVA, Tukey’s m.c.t.) (Fig. 3f).

Despite the reduction observed in P62 and LAMP-1 fluorescence levels following HFS in A53T α-Syn, their levels remained elevated when compared to pcDNA stimulated cells [P62 (30.81 ± 1.69 RFU A53T stimulated *vs* 15.7 ± 3.10 RFU pcDNA stimulated, ***p < 0.001, one-way ANOVA, Tukey’s m.c.t.) (Fig. 3b); LAMP-1 (24.62 ± 3.5 RFU A53T stimulated *vs* 13.7 ± 1.20 RFU pcDNA stimulated, *p < 0.05, one-way ANOVA, Tukey’s m.c.t.) (Fig. 3e)]. From these results, we infer that HFS only partially rescues autophagy impairment induced by mutant α-Syn.

### α-Syn mediated mitophagy upregulation is reduced by high frequency stimulation

 Since HFS partially restores α-Syn-mediated autophagy dysfunction, we explored whether HFS may modulate α-Syn-mediated dysfunction of mitochondrial autophagy. Mitophagy is a process in which damaged or aged mitochondria are selectively removed and degraded via the lysosomal system^58^. Dysregulation of mitophagy has been implicated in PD pathogenesis^59,60^ and was recently identified as a potential molecular target of STN-DBS following MPTP administration in mice^62^. The precise role of α-Syn on mitophagy remains poorly defined, with some studies demonstrating that α-Syn can inhibit mitophagy^59,61–63^ and others revealing its capacity to induce mitophagy^64–66^. We recently demonstrated that overexpression of A53T α-Syn leads to increased mitophagy in SH-SY5Y cells, primary cortical neurons, and in vivo. Further, we found that this phenomenon precedes dopaminergic degeneration^67^, highlighting the importance of restoring mitophagy imbalance to mitigate neurodegeneration^68^.

**Figure 4:**
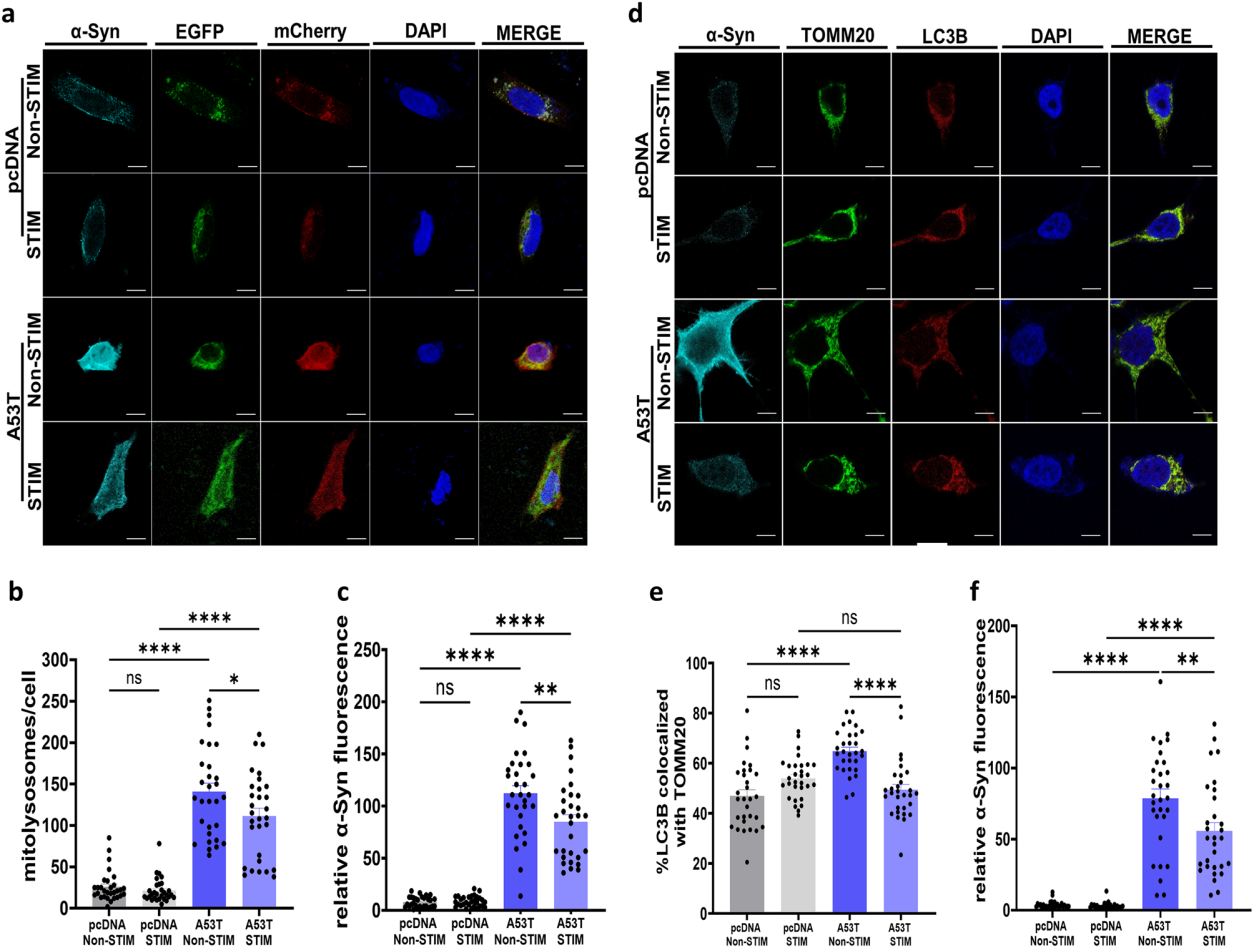
α-Syn mediated mitophagy upregulation is reduced by high frequency stimulation. (**a**) Representative images of SH-SY5Y cells transfected with mito-QC in combination of either A53T α-Syn or pcDNA as control, after stimulation for 2 h and non-stimulated controls, stained with DAPI (blue), anti-α-Syn (cyan), mCherry (red), and EGFP (green) (Scale bar 5 μm). (**b**) Quantification of mito-lysosomes/cell. Bars represent mean ± s.e.m., *p < 0.05, ****p < 0.0001, ns non-significant, n = 3 independent experiments, analyzing 10 cells per condition; one-way ANOVA, Tukey’s m.c.t. (**c**) Quantification of α-Syn fluorescence levels. Bars represent mean ± s.e.m., **p < 0.005, ****p < 0.0001, ns non-significant, n = 3 independent experiments, analyzing 10 cells per condition, one-way ANOVA, Tukey’s m.c.t. (**d**) Representative images of SH-SY5Y cells with either A53T α-Syn or pcDNA as control, after stimulation and non-stimulated controls, stained with DAPI (blue), anti-α-Syn (cyan), LC3B (red), and TOMM20 (green) (Scale bar 5 μm). (**e**) Quantification of LC3B puncta co-localized with TOMM20 in percentage, n = 3 independent experiments, analyzing 10 cells per condition. Bars represent mean ± s.e.m., ****p < 0.0001, ns non-significant,  one-way ANOVA, Tukey’s m.c.t. (**f**) Quantification of α-Syn fluorescence levels., n = 3 independent experiments, analyzing 10 cells per condition. Bars represent mean ± s.e.m., **p < 0.005, ****p < 0.0001, one-way ANOVA, Tukey’s m.c.t.

To determine the effects of HFS on α-Syn mediated mitophagy, we co-expressed A53T α-Syn or pcDNA as a control, along with the pH-sensitive mito-QC mitophagy reporter in SH-SY5Y cells (Fig. 4a). The mito-QC reporter is a binary-based fluorescence reporter, which co-localizes to the outer mitochondrial membrane and generates a readily quantifiable red signal when engulfed by an acidic lysosome^67,69^. We found that HFS partially normalized the number of red only puncta (mito-lysosomes) (111.3 ± 9.35 mito-lysosomes/cell stimulated *vs* 140.9 ± 10 mito-lysosomes/cell non-stimulated, *p < 0.05, one-way ANOVA, Tukey’s m.c.t.) (Fig. 4b). However, this reduction was not a complete rescue as the number of mito-lysosomes per cell was still higher in A53T stimulated cells than pcDNA stimulated controls (111.3 ± 9.35 mito-lysosomes/cell A53T stimulated *vs* 21.4 ± 2.6 mito-lysosomes/cell pcDNA stimulated ****p < 0.0001, one-way ANOVA, Tukey’s m.c.t.) (Fig. 4b), possibly due to α-Syn accumulation in stimulated cells (84.98 ± 6.7 RFU A53T stimulated *vs* 8.6 ± 0.9 RFU pcDNA stimulated, ****p < 0.0001, one-way ANOVA, Tukey’s m.c.t.) (Fig. 4c). We further assessed the effect of HFS on mitochondrial autophagy by analyzing the co-localization of  LC3 positive autophagosomes and TOMM20-labeled mitochondria in A53T α-Syn transfected SH-SY5Y cells (Fig. 4d). In line with our findings with the mito-QC reporter, HFS reduced co-localization (49.4 ± 2.1% co-localization A53T stimulated *vs* 64.8 ± 1.5% co-localization A53T non-stimulated, ****p < 0.0001, one-way ANOVA, Tukey’s m.c.t.) (Fig. 4e), indicating reduction in mitochondria engulfment by the lysosomes for degradation. This was in conjunction with a reduction in α-Syn levels (55.7 ± 5.9 RFU A53T stimulated *vs* 78.6 ± 6.6 RFU A53T non-stimulated, **p < 0.005, one-way ANOVA, Tukey’s m.c.t.) (Fig. 4f). Thus, HFS reduces α-Syn-mediated derangements in mitophagy.

### High frequency electrical stimulation reduces autophagy impairment due to bafilomycin

**Figure 5:**
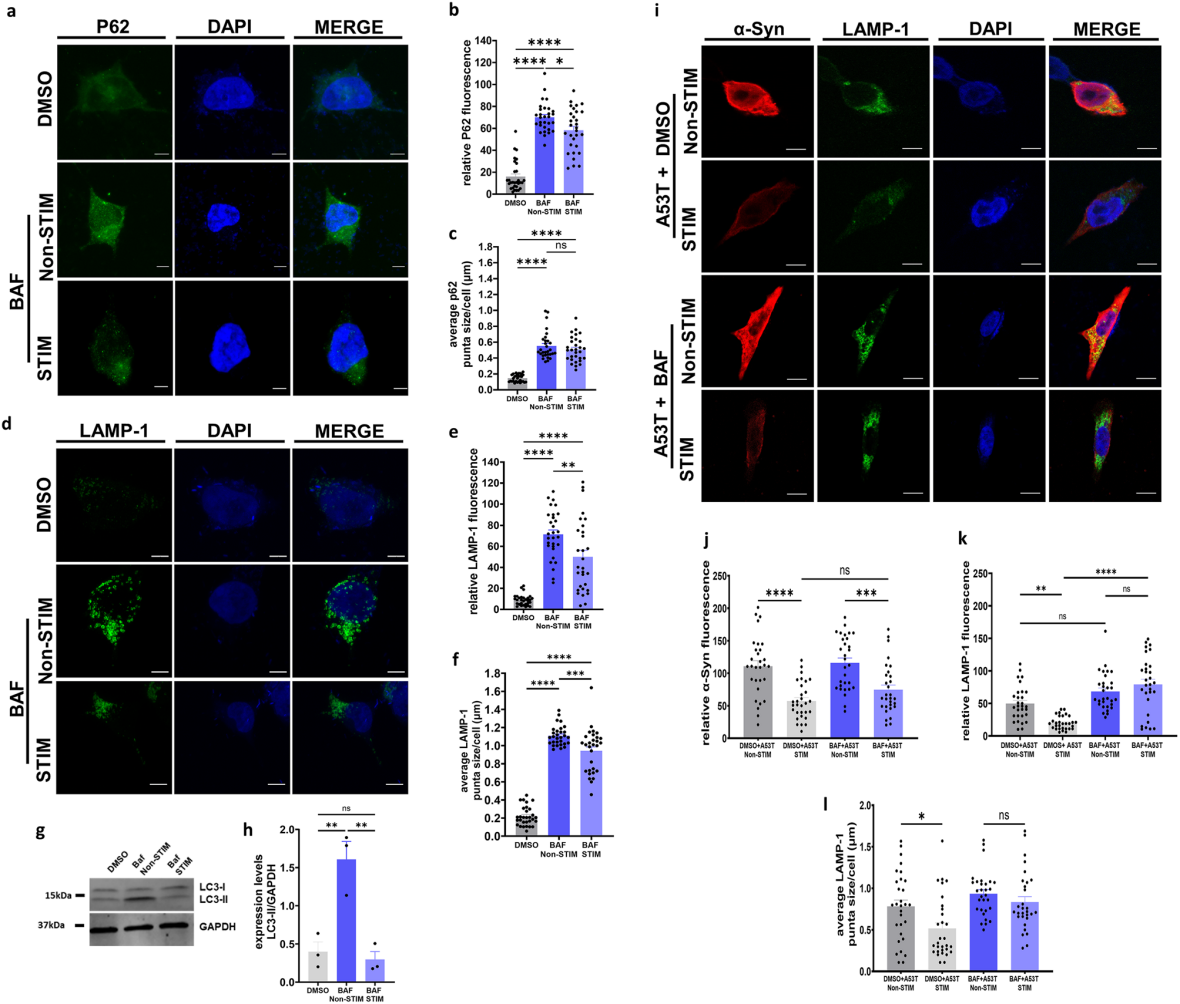
High frequency electrical stimulation reduces autophagy impairment due to bafilomycin. (**a**) Representative images of SH-SY5Y cells transfected with A53T or pcDNA after stimulation for 2 h and non-stimulated controls, stained with DAPI (blue), α-Syn (red) and P62 (green) (Scale bars 5 µm) (**b**) Quantification of P62 fluorescence. n = 3 independent experiments, analyzing 10 cells per condition. Bars represent mean ± s.e.m., *p < 0.05, ****p < 0.0001 one-way ANOVA, Tukey’s m.c.t. (**c**) Quantification of P62 average puncta size. n = 3 independent experiments, analyzing 10 cells per condition. Bars represent mean ± s.e.m., ****p < 0.0001, ns non-significant, one-way ANOVA, Tukey’s m.c.t. (**d**) Representative images of SH-SY5Y cells treated with BAF or DMSO controls after stimulation for 2 h and non-stimulated controls, stained with DAPI (blue) and LAMP-1 (green) (Scale bars = 5 µm) (**e**) Quantification of LAMP-1 fluorescence. n = 3 independent experiments, analyzing 10 cells per condition. Bars represent mean ± s.e.m., **p < 0.01, ****p < 0.0001, one-way ANOVA, Tukey’s m.c.t. (**f**) Quantification of LAMP-1 average puncta size. n = 3 independent experiments, analyzing 10 cells per condition. Bars represent mean ± s.e.m., ***p < 0.001, ****p < 0.0001, one-way ANOVA, Tukey’s m.c.t. (**g**) Western blot analysis of LC3-I and LC3-II in cells from DMSO and non-stimulated and stimulated bafilomycin conditions. GAPDH denoting loading control. Blot images are cropped from same blot and grouped together. Blot details and full length images are included in supplementary figures (**h**) Quantification of western blot expression levels of LC3-II/GAPDH. Bars represent mean ± s.e.m., **p < 0.005, ns non-significant, one-way ANOVA, Tukey’s m.c.t. (**i**) Representative images of SH-SY5Y cells transfected with A53T and treated with BAF, after stimulation for 2 h, stained with DAPI (blue), α-Syn (red) and LAMP-1 (green) (Scale bars = 5 µm) (**j**) Quantification of α-Syn fluorescence. n = 3 independent experiments, analyzing 10 cells per condition. Bars represent mean ± s.e.m., ***p < 0.001, ns non-significant, student’s two tailed t-test. (**k**) Quantification of LAMP-1 fluorescence. n = 3 independent experiments, analyzing 10 cells per condition. Bars represent mean ± s.e.m., **p < 0.005, ****p < 0.001, ns non-significant, one-way ANOVA, Tukey’s m.c.t. (**l**) Quantification of average LAMP-1 puncta size. n = 3 independent experiments, analyzing 10 cells per condition. Bars represent mean ± s.e.m., *p < 0.05, ns non-significant, one-way ANOVA, Tukey’s m.c.t.

Evidence suggests that aggregation of α-Syn is a consequence of impaired ALP degradation and that autophagy activation could facilitate its clearance^70,71^. Inducing autophagy in PC12 cells with lithium has been found to reduce mutant α-Syn levels^72^. Furthermore, enhancing autophagy via inhibition of mTOR/P70S6K signaling has been shown to induce degradation of A53T α-Syn^73^, supporting the capability of autophagy to clear α-Syn. Interestingly, stimulation mimicking tDCS was recently shown to activate macroautophagy and reduce the accumulation of α-Syn in rotenone treated cells^33^. To reconcile whether the partial restoration of autophagy function following HFS stems from a reduction in α-Syn levels and/or enhancement of autophagy activity , we examined whether HFS can overcome pharmacological impairment of autophagy. For this, we performed HFS in cells treated with Bafilomycin A1 (BAF), a specific vacuolar-type H^+^-ATPase inhibitor frequently used to block autophagy by inhibiting the acidification of lysosomes^46,74,75^. We observed that HFS for 2 h significantly altered the levels of autophagy markers [P62 (Fig. 5a) and LAMP-1 (Fig. 5d)] affected by BAF treatment. HFS significantly reduced the fluorescence levels of P62 (58.3 ± 3.5 RFU BAF stimulated *vs* 70 ± 2.4 RFU BAF non-stimulated, *p < 0.05, one-way ANOVA, Tukey’s m.c.t.) (Fig. 5b), without altering the average puncta size per cell (0.50 ± 0.02 µm BAF stimulated *vs* 0.55 ± 0.03 µm BAF non-stimulated) (Fig. 5c). Compared to non-stimulated BAF treated cells, HFS mitigated the elevated LAMP-1 fluorescence levels (50 ± 6.2 RFU BAF stimulated *vs* 71.4 ± 4.1 RFU BAF stimulated **p < 0.005, one-way ANOVA, Tukey’s m.c.t.) (Fig. 5e) and average puncta size per cell (0.94 ± 0.04 µm BAF stimulated *vs* 1.1 ± 0.01 µm BAF non-stimulated, ***p < 0.001, one-way ANOVA, Tukey’s m.c.t.) (Fig. 5f). Additionally, HFS was able to modulate the expression levels of autophagy-related microtubule-associated proteins 1A/1B light chain 3B, or LC3, altered due to BAF treatment (Fig. 5g). LC3B is often correlated with dysfunction in autophagy^7,76,77^ and contains two subtypes: LC3-I is the cytosolic precursor of LC3-II, and LC3-II is a specific marker of phagophores and subsequent autophagosomes. Western blot analysis revealed that HFS normalized expression levels of LC3-II/GAPDH in response to BAF treatment (0.29 ± 0.10 BAF stimulated *vs* 1.67 ± 0.23 BAF non-stimulated, **p < 0.005, one-way ANOVA Tukey’s m.c.t.) (Fig. 5h).

With alterations in LAMP-1 and LC3, but not P62 (average puncta size), HFS may have less effect on autophagosomes (P62), but could influence the ALP further downstream by restoring the formation of autolysosomes (LAMP-1 and LC3) (Fig. 5**)**. This in turn could be responsible for the clearance of α-Syn, which subsequently leads to the decrease in P62 levels (Fig. 3).  To confirm if HFS could still influence α-Syn reduction through action of HFS on autophagy, despite blockade by BAF, we stimulated cells transfected with A53T α-Syn and then treated with BAF (Fig. 5i). We found that HFS reduced A53T α-Syn levels that were elevated in BAF treated cells (74.65 ± 7.12 RFU stimulated *vs* 115.9 ± 7.48 RFU non-stimulated, ***p < 0.001 one-way ANOVA, Tukey’s m.c.t.) (Fig. 5j). However, unexpectedly, HFS did not alter the elevated fluorescence levels (79.20 ± 7.92 RFU stimulated *vs* 68.29 ± 5.23 RFU non-stimulated) (Fig. 5k) or the average puncta size per cell (0.83 ± 0.064 µm stimulated *vs* 0.093 ± 0.049 µm non-stimulated) (Fig. 5l) of LAMP-1. This suggests that, while HFS can rescue general autophagy dysfunction sufficiently to reduce α-Syn, its beneficial effects are uncoupled from α-Syn-mediated autophagy impairment.

### High frequency electrical stimulation affects V-ATPase in SH-SY5Y cells

We observed that HFS can mitigate autophagy dysfunction induced by mutant α-Syn accumulation or by BAF treatment **(**Fig. 5a–h**)**. BAF causes inhibition of vacuolar-type H^+^-ATPases (V-ATPases), which are ATP-driven proton pumps responsible for maintaining the acidic pH of intracellular organelles, such as lysosomes^78–80^. The mammalian V‐ATPase is comprised of 13 distinct subunits, 8 of which are part of the cytosolic V1 (A-H) domain and 5 of which are part of the transmembrane V0 (a, c, c″, d and e) domain. ATP hydrolysis occurs within the V1 domain and drives rotation of the central stalk, resulting in H^+^‐transport via the V0 domain^75,81,82^. The complex is orientated such that protons are transported from the cytoplasm to the lumen of lysosomes. Thus, V-ATPases play an important role in autophagosome-lysosome fusion and autolysosome acidification, two processes disrupted by BAF treatment^74,75^. BAF binds to subunit c of the V0 domain (ATP6V0C), inhibiting the translocation of protons across the pump and thereby causing de-acidification of lysosomes^75,83,84^. With HFS partially restoring the effect on autophagy dysfunction caused by BAF, we hypothesized that HFS modulates this effect through ATP6V0C.

**Figure 6:**
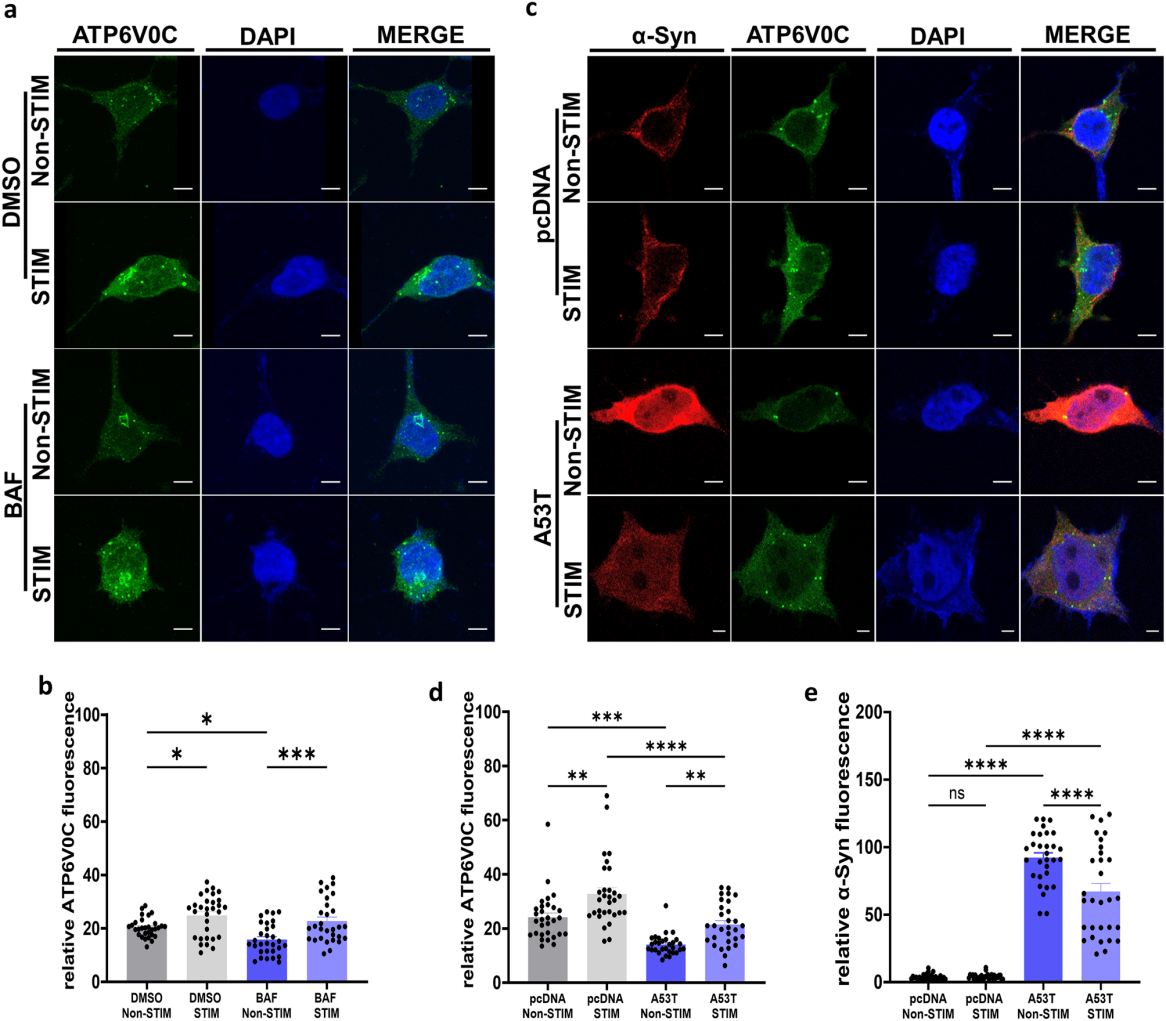
High frequency electrical stimulation affects V-ATPase in SH-SY5Y cells. (**a**) Representative images of SH-SY5Y cells treated with BAF or DMSO after stimulation for 2 h and non-stimulated controls, stained with DAPI (blue), α-Syn (red) and ATP6V0C (green) (Scale bars 5 µm) (**b**) Quantification of ATP6V0C fluorescence. n = 3 independent experiments, analyzing 10 cells per condition. Bars represent mean ± s.e.m., *p < 0.05, ***p < 0.001, one-way ANOVA, Tukey’s m.c.t. (**c**) Representative images of SH-SY5Y cells transfected with A53T or pcDNA after stimulation for 2 h and non-stimulated controls, stained with DAPI (blue), α-Syn (red) and ATP6V0C (green) (Scale bars 5 µm) (**d**) Quantification of ATP6V0C fluorescence levels. n = 3 independent experiments, analyzing 10 cells per condition. Bars represent mean ± s.e.m., *p < 0.05 **p < 0.005, ****p < 0.0001, one-way ANOVA, Tukey’s m.c.t. (**e**) Quantification of α-Syn fluorescence levels. n = 3 independent experiments, analyzing 10 cells per condition. Bars represent mean ± s.e.m., ****p < 0.0001, ns non-significant, one-way ANOVA, Tukey’s m.c.t.

We performed HFS on SH-SY5Y cells treated with BAF and examined levels of ATP6V0C (Fig. 6a). Cells treated with BAF showed reduced expression levels of ATP6V0C compared to DMSO vehicle controls (15.83 ± 1 RFU BAF *vs* 24 ± 1.6 RFU DMSO, *p < 0.05, one-way ANOVA, Tukey’s m.c.t.) (Fig. 6b). HFS enhanced the fluorescence levels of ATPV0C in BAF treated cells (22.73 ± 1.4 RFU stimulated *vs* 15.83 ± 1 RFU non-stimulated, ***p < 0.001, one-way ANOVA, Tukey’s m.c.t.) (Fig. 6b). Additionally, HFS increased ATP6V0C fluorescence levels in DMSO treated cells (32.7 ± 2.2 RFU stimulated *vs* 24 ± 1.6 non-stimulated, *p < 0.05, one-way ANOVA, Tukey’s m.c.t.) (Fig. 6b), suggesting that HFS affects ATP6V0C regardless of the conditions.

ATP6V0C has been implicated in PD. For instance, reduced expression of ATP6V0C was observed in the SNpc of PD brains compared to healthy controls^85^. Overexpression of ATP6V0C in the SNpc of mice was found to promote dopamine release in the striatum and improve motor performance in 6-OHDA-lesioned mice administered DA-synthesizing enzymes^86^. Furthermore, ATP6V0C knockdown in SH-SY5Y cells promoted the accumulation of high molecular weight species of α-Syn, in addition to increasing basal levels of LC3-II diminishing autophagic flux^87^, hinting towards a possible causal relationship between α-Syn and ATP6V0C with regards to autophagy dysfunction. Corroborating this theory, we observed that SH-SY5Y cells transfected with A53T α-Syn had reduced levels of ATP6V0C compared to pcDNA transfected controls (14 ± 0.6 RFU A53T *vs* 24.09 ± 1.6 RFU pcDNA, ***p < 0.001 one-way ANOVA, Tukey’s m.c.t.) (Fig. 6c, d), suggesting that accumulation of α-Syn impacts ATP6V0C. To investigate whether HFS-mediated reduction of α-Syn modulated ATP6V0C levels, we stimulated pcDNA and A53T α-Syn transfected cells. HFS enhanced ATP6V0C fluorescence levels of pcDNA transfected cells compared to non-stimulated pcDNA controls (32.7 ± 2.2 RFU stimulated *vs* 24.09 ± 1.6 RFU non-stimulated, **p < 0.005, one-way ANOVA, Tukey’s m.c.t.) and of A53T α-Syn transfected cells (20.9 ± 1.4 RFU stimulated *vs* 14 ± 0.6 RFU non-stimulated, **p < 0.005, one-way ANOVA, Tukey’s m.c.t.) (Fig. 6d). Although HFS similarly increased ATP6V0C in A53T α-Syn transfected cells, levels of the ATPase remained lower than in pcDNA-transfected stimulated cells (20.9 ± 1.4 RFU A53T stimulated *vs* 32.7 ± 2.2 RFU pcDNA stimulated, ****p < 0.001, one-way ANOVA, Tukey’s m.c.t.) (Fig. 6d), indicating a partial rescue. The increase in ATP6V0C due to HFS occurred in conjunction with reduced levels α-Syn (72.2 ± 8.1 RFU stimulated *vs* 92.2 ± 3.5 RFU non-stimulated, ****p < 0.05, one-way ANOVA, Tukey’s m.c.t.) (Fig. 6e), and thus the effects of HFS on ATP6V0C may cause reduction in α-Syn levels.

### High frequency electrical stimulation reduces α-Syn levels and α-Syn-mediated autophagy dysfunction in neurons

**Figure 7:**
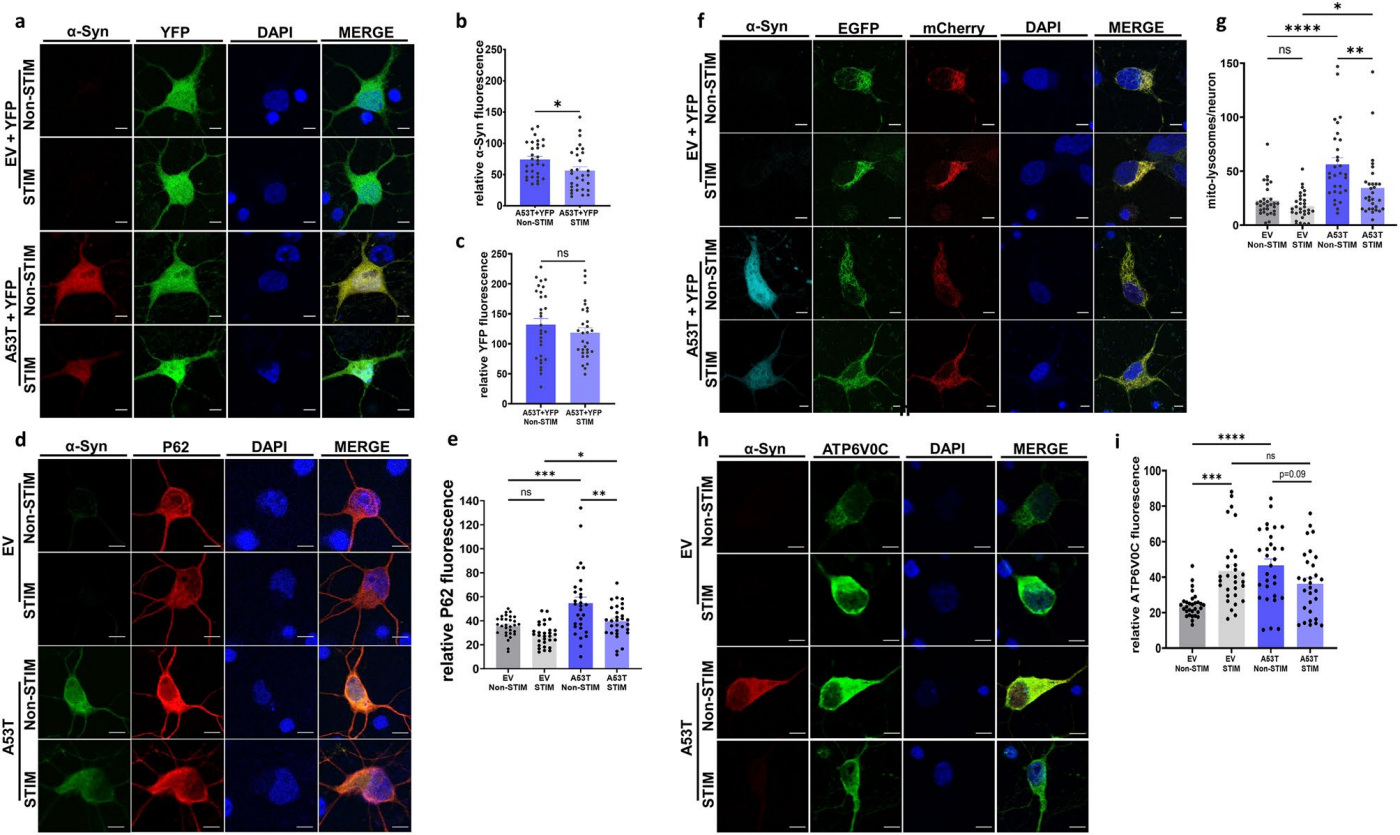
High frequency electrical stimulation reduces α-Syn levels and α-Syn-mediated autophagy dysfunction in neurons. (**a**) Representative confocal images of primary cortical neurons co-transduced with AAV1/2 expressing mutant A53T α-Syn or empty vector (EV), along with YFP, after stimulation and non-stimulated controls, stained using DAPI (blue), anti-α-Syn (red) and YFP (green) (Scale bars 5 µm). (**b**) Quantification of relative α-Syn fluorescence levels. Bars represent mean ± s.e.m., *p < 0.05. n = 3 independent experiments, analyzing 10 cells per condition. Two-tailed unpaired t-test (**c**) Quantification of relative YFP fluorescence. Bars represent mean ± s.e.m., ns non-significant, n = 3 independent experiments, analyzing 10 cells per condition. Two-tailed unpaired t-test (**d**) Representative images of cortical neurons infected with A53T or EV after stimulation, stained with DAPI (blue), α-Syn (green) and P62 (red) (Scale bars 5 µm). (**e**) Quantification of P62 fluorescence levels. Bars represent mean ± s.e.m., **p < 0.005, ***p < 0.001., n = 3 independent experiments, analyzing 10 cells per condition, one-way ANOVA, Tukey’s m.c.t. (**f**) Representative images of cortical neurons infected with viruses expressing mito-QC with either HA-tagged A53T α-Syn or EV. Nuclear counter staining with DAPI (blue), anti-α-Syn (cyan), mCherry (red), and EGFP (green), after stimulation and non-stimulated controls (Scale bars 5 µm). (**g**) Quantification of mito-lysosomes. Bars represent mean ± s.e.m., *p < 0.05, **p < 0.005, ****p < 0.0001. n = 3 independent experiments, analyzing 10 cells per condition, one-way ANOVA, Tukey’s m.c.t. (**h**) Representative images of cortical neurons infected with A53T or EV after stimulation, stained with DAPI (blue), α-Syn (red) and ATP6V0C (green) (Scale bars 5 µm) (**i**) Quantification of ATP6V0C fluorescence levels. Bars represent mean ± s.e.m., *** p < 0.001, ****p < 0.0001, ns non-significant. n = 3 independent experiments, analyzing 10 neurons per condition, one-way ANOVA, Tukey’s m.c.t.

Having demonstrated the effects of HFS on α-Syn levels and on α-Syn-mediated dysfunction in SH-SY5Y cells, we aimed to replicate key findings in neurons. To achieve this, we cultured primary rat cortical neurons, transduced them with A53T α-Syn or empty vector (EV) AAVs, and used the same HFS parameters^32^. Neurons were co-transduced with YFP as an internal control to assess for non-specific effects of HFS. Corroborating our findings in SH-SY5Y cells, HFS decreased fluorescence levels of A53T α-Syn in stimulated neurons compared to non-stimulated A53T α-Syn transduced neurons (56.38 ± 6.2 RFU stimulated *vs* 74.0 ± 5.0 RFU non-stimulated, *p < 0.05, one-way ANOVA, Tukey’s m.c.t.) (Fig. 7a,b) with no differences in YFP fluorescence intensity observed (118.4 ± 8.3 RFU stimulated *vs* 131.8 ± 10.4 RFU non-stimulated) (Fig. 7c).

We first examined whether the reduction in α-Syn levels observed in neurons impacts α-Syn-mediated autophagy dysfunction (Fig. 7d). A53T α-Syn overexpression was associated with an increase in P62 fluorescence levels compared to EV transduced neurons (54.62 ± 5.04 RFU A53T *vs* 35.51 ± 1.58 RFU EV, ***p < 0.001, one-way ANOVA, Tukey’s m.c.t.) (Fig. 7e). HFS led to a partial reduction in the fluorescence levels of P62 in A53T α-Syn transduced neurons (39.63 ± 2.50 RFU stimulated *vs* 54.62 ± 5.04 RFU non-stimulated, **p < 0.005, one-way ANOVA, Tukey’s m.c.t.) (Fig. 7e).

We next assessed for a possible mitigating effect of HFS on mitophagy by using the AAV vector of mitoQC^67^ (Fig. 7f). Rat primary cortical neurons were co-transduced with AAV-A53T or AAV-EV plus AAV-mitoQC. Based on the quantification of red only puncta (mito-lysosomes) within each neuron. HFS reduced the number of mito-lysosomes/cell (34.37 ± 5.1 mito-lysosomes/cell stimulated *vs* 56.2 ± 6.2 mito-lysosomes/cell non-stimulated, **p < 0.005, one-way ANOVA, Tukey’s m.c.t.) (Fig. 7g). This number was still significantly higher when compared to EV stimulated neurons (34.37 ± 5.1 mito-lysosomes/cell A53T stimulated 17.57 ± 2.1 mito-lysosomes/cell pcDNA stimulated, *p < 0.05, one-way ANOVA, Tukey’s m.c.t.) (Fig. 7g), indicating that HFS partially rescues α-Syn-mediated mitophagy impairments in neurons.

Finally, we investigated whether HFS affects ATP6V0C in neurons (Fig. 7h). We observed that A53T α-Syn overexpression was associated with an elevation in ATP6V0C fluorescence levels compared to EV controls (46.59 ± 3.66 RFU A53T *vs* 24.27 ± 1.25 RFU EV, ****p < 0.0001, one-way ANOVA, Tukey’s m.c.t.) (Fig. 7i). Similar to our observations in SH-SY5Y cells, HFS enhanced ATP6V0C fluorescence levels in EV transduced cells compared to non-stimulated controls (43.54 ± 3.57 RFU stimulated *vs* 24.27 ± 1.25 RFU non-stimulated, ***p < 0.001, one-way ANOVA, Tukey’s m.c.t.) (Fig. 7i). HFS did not affect the increased ATP6V0C fluorescence levels observed in A53T α-Syn transduced neurons (36.29 ± 3.31 RFU stimulated *vs* 46.59 ± 3.66 RFU non-stimulated) (Fig. 7i). This suggests that, unlike our observations in SH-SY5Y cells, the  reduction in α-Syn with HFS in neurons is not associated with increases in ATP6V0C.

## Discussion

Our results illustrate that HFS, mimicking DBS, limits the accumulation of mutant A53T α-Syn and its derangements on cellular pathways involved in the clearance of misfolded proteins and damaged organelles. As part of our previous study^32^, we showed that HFS can decrease levels of mutant α-Syn and WT α-Syn oligomers in transduced cortical neurons stimulated for 3 h. However, the pathway responsible for this reduction was not deciphered. Using the same HFS parameters, we found here that reduction in levels of A53T α-Syn in SH-SY5Y cells and in cortical neurons can occur in as little as 2 h. Furthermore, we found this effect to be stable for short periods of time and transient over longer periods. The levels of α-Syn remained lower 3 h after the end of stimulation and then reverted to that of non-stimulated controls 24 h after the end of stimulation. Moreover, there were no differences in α-Syn mRNA levels between stimulated and non-stimulated A53T α-Syn or pcDNA cells, implying that the transient decrease observed was not due to HFS modifying transcription of α-Syn but due to it enhancing clearance of the accumulated protein.

Prior evidence implicates both UPS^88,89^ and ALP^5,47,72,73,90^ as pathways responsible for α-Syn degradation. Moreover, dysfunction of these pathways is associated with α-Syn accumulation and implicated in the neurodegenerative process in PD^9,12,46,71^. We found that overexpression of A53T α-Syn in SH-SY5Y cells caused an increase in markers associated with UPS and autophagy dysfunction. We also found that a reduction in α-Syn levels due to HFS was associated with improvements in UPS and autophagy dysfunction in both SH-SY5Y cells and neurons. Furthermore, we demonstrated that in both SH-SY5Y cells and neurons HFS led to a decrease of mitophagy, a process that precedes neurodegeneration^67^. Thus, our observations that HFS reduces not only α-Syn, but also dysfunction in UPS, autophagy, and mitophagy, highlight its therapeutic potential in mitigating the detrimental impact of α-Syn on neurons in multiple manners.

To identify the pathways involved in HFS-mediated α-Syn degradation, we employed the use of pharmacological inhibitors. We observed that HFS failed to alter the increased Ub^G76V^-GFP levels following MG132-induced proteasome impairment. Unlike that observed in A53T α-Syn transfected cells, HFS was unable to mitigate the elevated levels of Ub^G76V^-GFP in A53T α-Syn cells treated with MG132, despite reduced α-Syn levels with HFS, signalling that HFS clearance of α-Syn was not dependent on the UPS. Similarly, the autophagy inhibitor BAF did not inhibit the clearance of α-Syn by HFS, which would potentially discount the ALP as a target for electrical stimulation as well. However, we found that HFS reduces autophagy dysfunction due to BAF treatment alone and thus does impact the ALP.

With HFS reducing autophagy dysfunction due to BAF, we focused our attention on BAF’s site of action – ATP6V0C – as a potential mechanism for HFS. We analyzed the expression of ATP6V0C which has previously been shown be decreased in PD patient brains^85^. Blockade of ATP6V0C inhibits the acidification of lysosomes and interferes with the ALP, thus preventing degradation and promoting accumulation of aggregated proteins, such as α-Syn^87,91^. Supporting our hypothesis, we found that cells transfected with A53T α-Syn exhibited lower levels of ATP6V0C that could be partially restored by short-term HFS. Furthermore, HFS also restored levels of ATP6V0C following BAF treatment. Although the mechanism underlying the activation of ATP6V0C is unclear, studies have shown that ATP hydrolysis can be differentially modulated by the frequency of oscillating electric fields of sine waveform^92,93^. Increased ATP hydrolysis by V-ATPase leads to influx of protons across the pump, enabling the acidification of lysosomes and subsequent protein degradation by pH-sensitive proteases. Although the electric fields generated in our study were of square waveform, in which the amplitude alternates at a steady frequency between fixed minimum and maximum values, the potential ability of HFS to stimulate V-ATPase to reduce autophagy dysfunction remains a consideration. In vitro DCS has been shown to influence autophagy and oligomeric/aggregated forms of α-Syn while increasing soluble monomeric α-Syn; the most robust effect was observed 17 h from end of stimulation^33^. Notwithstanding the differences in stimulation protocols and experimental parameters, ours is the first study to implicate V-ATPase, specifically ATP6V0C, as a potential site of action for electrical stimulation in models of α-Syn accumulation and α-Syn-mediated cellular dysfunction.

In our study, electrical stimulation caused significant reduction of accumulated α-Syn, but the reduction was not complete and was transient. Electrical stimulation also only partially rescued UPS and autophagy dysfunction. Further research to understand the optimal frequency, pulse width, and current settings sufficient for robust and prolonged reduction in α-Syn and α-Syn-mediated UPS and autophagy impairment is needed.

Our study is not without limitations. Although we propose that HFS targets ATP6V0C in SH-SY5Y cells, the ability of HFS to mitigate the altered levels of ATP6V0C associated with A53T α-Syn overexpression was not observed in neurons, despite ATP6V0C levels being reduced with HFS in EV conditions. Mutant α-Syn overexpression in neurons likely affects more than just the ATP6V0C subunit. Further exploration of other ATPase subunits is needed to confirm the specificity of the action of HFS. Moreover, it is important to note our study involves short-term stimulation and only mutant A53T α-Syn. As such, future studies will be required to assess the mechanistic underpinnings of these changes in other forms of α-Syn (e.g., WT, oligomers, fibrils). Although recapitulating DBS using HFS in vitro to understand the effects on α-Syn and associated cellular dysfunction is feasible, future preclinical studies will be needed to create effective translational paths to explore repurposing DBS for people with PD.

### Supplementary Information

Supplementary Information.

## Data Availability

All data generated or analyzed during this study are included or cited in this published article and its supplementary information files.
