# Supplementary material for: High frequency electrical stimulation reduces α-synuclein levels and α-synuclein-mediated autophagy dysfunction
Source: Sci Rep. 2024 Jul 12;14:16091. doi: 10.1038/s41598-024-64131-3 (PMC11245498; doi:10.1038/s41598-024-64131-3)
Supplement: Supplementary file 1 — Supplementary Information. [file 41598_2024_64131_MOESM1_ESM.pdf]

HIGH FREQUENCY ELECTRICAL STIMULATION REDUCES α-SYNUCLEIN LEVELS AND α-SYNUCLEIN-MEDIATED AUTOPHAGY DYSFUNCTION

Jimmy George, Kashfia Shafiq, Minesh Kapadia, Lorraine V. Kalia and Suneil K. Kalia

SUPPLEMENTARY FIGURES

Fig S1. Western blots from SH-SY5Y cells treated with Bafilomycin A1 (40 nM) with and without HFS.

Panel A: A full-length PVDF membrane of LC3-I and LC3-II bands with molecular weight ladders; cropped images of LC3-I and LC3-II bands indicated by the red box shown in Fig. 5g.

Panel B: The membrane in Panel A was stripped and exposed to anti-GAPDH; cropped images of GAPDH bands indicated by the red box shown in Fig. 5g.

A

1 2 3 4 5 6

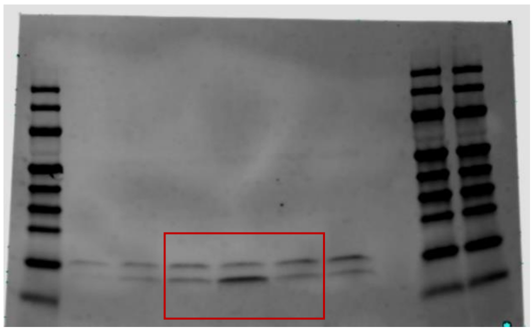

B

1 2 3 4 5 6

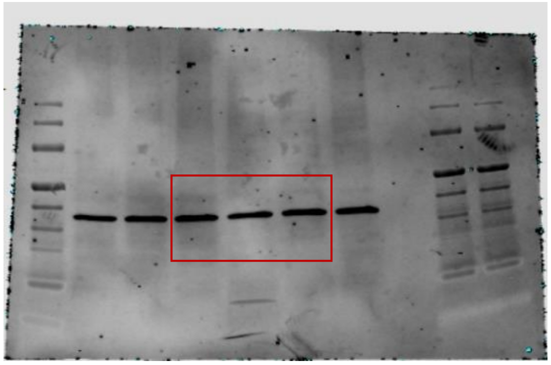

1. DMSO
2. DMSO
3. DMSO
4. BAF Non-stimulated
5. BAF Stimulated
6. BAF Stimulated
